# Poor outcomes of immunoglobulin D multiple myeloma patients in the era of novel agents: a single-center experience

**DOI:** 10.1186/s40880-019-0395-3

**Published:** 2019-09-27

**Authors:** Qian Zhao, Feng Li, Ping Song, Xiaogang Zhou, Zhiming An, Jiangang Mei, Jingjing Shao, Hanqing Li, Xuli Wang, Xing Guo, Yongping Zhai

**Affiliations:** Department of Hematology, Jinling Hospital, School of Medicine, Nanjing University, 305 East Zhongshan Road, Xuanwu District, Nanjing, 210002 Jiangsu P. R. China

Dear Editor,

Multiple myeloma (MM) is a disease characterized by the clonal expansion of malignant plasma cells in the marrow, leading to anemia, hypercalcemia, bone lesion, and renal dysfunction [1]. Immunoglobulin D (IgD) myeloma is a rare subtype of MM, accounting for approximately 1% to 2% of all MM patients [2]. It occurs at a young age, often accompanied with a high disease burden and short median survival (18–21 months) [3, 4]. Several studies have suggested that in patients with IgD subtype, the outcomes of those who have had undergone autologous stem cell transplantation (ASCT) were superior than those treated with chemotherapy alone [5, 6]. However, these have been debatable as other reports have displayed opposite results [7, 8].

In the last decade, novel agents such as proteasome inhibitor and immunomodulatory agents have been used to treat MM, which have resulted in a 50% improvement in the patients’ overall survival (OS: 44.8 vs. 29.9 months) [9]. However, given the rarity of IgD myeloma, studies on it remain rare. Knowledge about this subtype was mostly derived from few single-center case series. In China, there has been no report to fully elucidate on whether the results of survival outcomes for patients with IgD subtype from novel agents could be replicated in those with non-IgD subtypes. Here, we performed a retrospective analysis on 216 symptomatic MM patients, diagnosed from August 2006 to April 2018, at the Jinling Hospital (Nanjing, Jiangsu, China). The patient cohort comprised of 13 with IgD subtype and 203 with non-IgD subtypes. Three new drugs, bortezomib, thalidomide, and lenalidomide, were used as induction therapy choice, based on which we investigated their clinical manifestations, treatment responses, and outcomes. In this study cohort, 1 patient with IgD subtype and 11 with non-IgD subtypes underwent ASCT.

First, we compared the clinical features between the IgD and non-IgD subtypes (Table 1). The median age of onset for the IgD subtype was 52 years, which was younger than that of the non-IgD subtypes (60 years, *P* = 0.028). The rate of λ light chain was significantly higher in patients with the IgD subtype than in those with non-IgD subtypes (92.3% vs. 48.8%, *P* = 0.006). Moreover, as compared with non-IgD subtypes, patients with IgD subtype presented more often with significant renal dysfunction (creatinine > 2 mg/L, *P* < 0.001) and amyloid light-chain (AL) amyloidosis (*P* = 0.001) and had higher frequencies of critical clinical features such as International staging system (ISS)-III disease (*P* = 0.002), severe anemia (Hemoglobin < 90 g/L, *P* = 0.011), high β_2_ microglobulin (β_2_Μ) concentration (*P* = 0.001), and elevated lactate dehydrogenase (LDH > 250 U/L, *P* = 0.002). However, there were no significant differences in sex, platelet counts, bone lesion, hypercalcemia, urine protein, induction therapy, and extramedullary infiltration between IgD and non-IgD subtypes.

**Table 1:** Clinical characteristics and treatment of 216 patients with multiple myeloma

Characteristics	IgD subtype	Non-IgD subtypes	*P* value
Cases, *n*	13	203	
Age [years; median (range)]	52 (44–66)	60 (24–82)	0.028
Sex [cases (%)]			0.782
Male	8 (61.5)	117 (57.3)	
Female	5 (38.5)	86 (42.7)	
Hemoglobin [g/L; mean (range)]	73 (44–108)	90 (40–107)	0.011
Platelets < 100 × 10^9^/L [cases (%)]	4 (30.8)	32 (15.8)	0.306
Light chain type [cases (%)]			0.006
κ	1 (7.7)	104 (51.2)	
λ	12 (92.3)	99 (48.8)	
Creatinine > 2 mg/dL [cases (%)]	9 (69.2)	47 (23.2)	< 0.001
β_2_M [mg/L; mean (range)]	10 (2.57–16.50)	5.16 (1.45–19.40)	0.001
Bone lesion [cases (%)]	10 (76.9)	170 (83.7)	0.459
Hypercalcemia [cases (%)]	3(23.1)	14 (6.9)	0.117
Urine protein (g/L)	3.20 (0.15–19.69)	1.30 (0.10–26.10)	0.196
LDH > 250 U/L [cases (%)]	7 (53.8)	37 (18.2)	0.002
ISS stage [cases (%)]			0.002
I	0	41 (20.2)	
II	0	58 (28.6)	
III	13 (100.0)	104 (50.2)	
Induction therapy [cases (%)]			0.080
IMiD-based regimens	4 (30.8)	121(59.6)	
Bortezomib-based regimens	9 (69.2)	82 (40.4)	
Final response [cases (%)]			0.847
sCR, CR	4 (30.8)	68 (33.5)	
VGPR	3 (23.1)	80 (39.4)	
PR	4 (30.8)	43 (20.9)	
SD plus PD	2 (15.4)	12 (5.8)	
AL amyloidosis [cases (%)]	4 (30.8)	8 (3.9)	0.001
Extramedullary infiltration [cases (%)]	2 (15.4)	57 (28.1)	0.500

*IgD* immunoglobulin D, *β*_*2*_*M* β_2_ microglobulin, *LDH* lactate dehydrogenase, *ISS* international staging system, *IMiD* immunomodulatory drug, *sCR* strict complete response, *CR* complete response, *VGPR* very good partial response, *PR* partial response, *SD* stable disease, *PD* progressive disease, *AL* amyloidosis amyloid light-chain amyloidosis

Based on the data from Table 2, we found that 30.8% (4/13) of patients with IgD subtype presented with significant abnormalities in serum-free light chain (sFLC) ratio (< 0.01 or > 100) at baseline, compared with 7.5% (13/173) for the non-IgD subtypes (*P *= 0.021). Moreover, about half of those (46.2%) with IgD subtype showed an obvious increase in sFLC ratio abnormalities when relapsed (*P *< 0.001).

**Table 2:** The sFLC concentrations and abnormal ratios of the investigated 186 patients

Variable	Baseline			After relapse		
	IgD subtype (*n* = 13)	Non-IgD subtypes (*n *= 173)^a^	*P* value	IgD subtype (*n *= 13)	Non-IgD subtypes (*n *= 96)	*P* value
sFLC [mg/L; median (range)]						
κ	12.10 (8.54–186.40)	25.80 (1.49–17,780.00)	0.567	15.60 (8.50–1708.00)	56.00 (7.49–4541.00)	0.655
λ	129.70 (8.46–4303.00)	28.80 (1.33–17,600.00)	0.151	583.86 (10.51–1092.00)	687.00 (1.33–4000.00)	0.412
κ/λ < 0.01 or > 100 [cases (%)]	4 (30.78)	13 (7.51)	0.021	6 (46.15)	7 (7.29)	< 0.001

*sFLC* serum free light chain, *IgD* immunoglobulin D

^a^Serum-free light chains were only available for 173 cases of MM patients at the time of diagnosis

Of the 216 MM patients, 198 (91.7%) were subjected to cytogenetic abnormality testing (Table 3). 1q21 amplification was discovered in 9 patients with IgD subtype, which was higher than that in patients with non-IgD subtypes (75.0% vs. 40.3%, *P *= 0.018). The rate of t (14;16) was also significantly higher in patients with IgD subtype than those with the non-IgD subtypes (17.0% vs. 1.6%, *P *= 0.023). However, no significant differences in other cytogenetic abnormalities such as 13 deletions, t (4;14), t (11;14), p53 deletion, and hyperdiploid between the IgD and non-IgD subtypes were found.

**Table 3:** Abnormal cytogenetic characteristics in 198 case of MM patients

Variable	IgD subtype [cases (%)]	Non-IgD subtypes [cases (%)]	*P* value
Total	12	186	
13 deletion	4 (33.3)	59 (31.7)	0.907
1q21 amplification	9 (75.0)	75 (40.3)	0.018
IGH rearrangement	6 (50.0)	59 (31.7)	0.191
t(4;14)	3 (25.0)	31 (16.7)	0.729
t(11;14)	1 (8.0)	25 (13.4)	0.866
t(14;16)	2 (17.0)	3 (1.6)	0.023
p53 deletion	0 (0)	7 (3.8)	1.000
Hyperdiploid	4 (33.3)	71 (38.2)	0.948

*IgD* immunoglobulin D, *IGH* immunoglobulin heavy chain gene

Second, we analyzed the treatment response between the IgD and non-IgD subtypes and have listed their detailed information regarding the treatment and survival of the patients with IgD subtype in Table 4. By the end of follow-up on August 31, 2018, 71 deaths were recorded of whom 9 cases (69.2%) were from patients having the IgD subtype. The overall response rate of the entire cohort was 93.5% (202/216). Response of induction therapy for the IgD subtype was similar to that of the non-IgD subtypes (*P *= 0.847, Table 1). However, the median duration of response in patients with IgD subtype was 10 months, which was significantly shorter than that of patients with non-IgD subtypes (23.6 months, *P *= 0.002) (Fig. 1a). The median follow-up of the 216 patients was 32.4 months (range 0.96–147 months). In patients with IgD subtype, the median progression-free survival (PFS) was 10.0 months and the median OS was 22.9 months, compared with 27.9 months (*P *= 0.003; Fig. 1b) and 81.7 months (*P *< 0.001; Fig. 1c) for patients bearing the non-IgD subtypes.

**Table 4:** Treatment and survival of the 13 patients with IgD subtype MM

Patient no.	ISS stage	Sex	Age (years)	Introduction therapy/cycles	Response	Therapy after PD/cycles	Survival status	PFS (months)	OS (months)
1	III	Male	51	CTD/5	CR	RAD/6	Alive	6.0	17.6
2	III	Female	53	CTD/7	PR	RD/8	Alive	8.0	19.5
3	III	Male	64	VCD/1	PD	NA	Dead	0.7	0.9
4	III	Male	48	VTD/8	PR	RCD	Dead	26.2	44.3
5	III	Female	59	VTD/2	SD	NA	Dead	5.1	5.7
6	III	Male	44	VCD/9	CR	TAD/6 + RAD/6	Alive	42.3	47.0
7	III	Female	49	VTD/9	CR	RAD/10	Dead	28.0	42.6
8	III	Male	52	VCD/9	VGPR	RD/6	Dead	8.5	13.0
9	III	Male	45	VCD/2	CR	CTD/8	Alive	2.4	9.2
10	III	Male	59	VD/4	PR	VCTD/5	Dead	16.7	16.7
11	III	Male	51	CTD/5	PR	RAD/3	Dead	10.0	6.6
12	III	Female	56	VCD/4 + ASCT	VGPR	CTD/4	Dead	18.7	22.9
13	III	Female	66	VD/4	VGPR	RD/6	Dead	48.0	66.0

*IgD* immunoglobulin D, *ISS* international staging system, *PD* progressive disease, *PFS* progression-free survival, *OS* overall survival, *CTD* thalidomide + cyclophosphamide + dexamethasone, *CR* complete response, *RAD* lenalidomide + adriamycin + dexamethasone, *PR* partial response, *RD* lenalidomide + dexamethasone, *VCD* bortezomib + cyclophosphamide + dexamethasone, *PD* progressive disease, *NA* not applicable, *VTD* bortezomib + thalidomide + dexamethasone, *RCD* lenalidomide + cyclophosphamide + dexamethasone, *SD* stable disease, *TAD* thalidomide + adriamycin + dexamethasone, *VGPR* very good partial response, *VD* bortezomib + dexamethasone, *VCTD* bortezomib + thalidomide + cyclophosphamide + dexamethasone, *ASCT* autologous stem cell transplantation

**Fig. 1:**
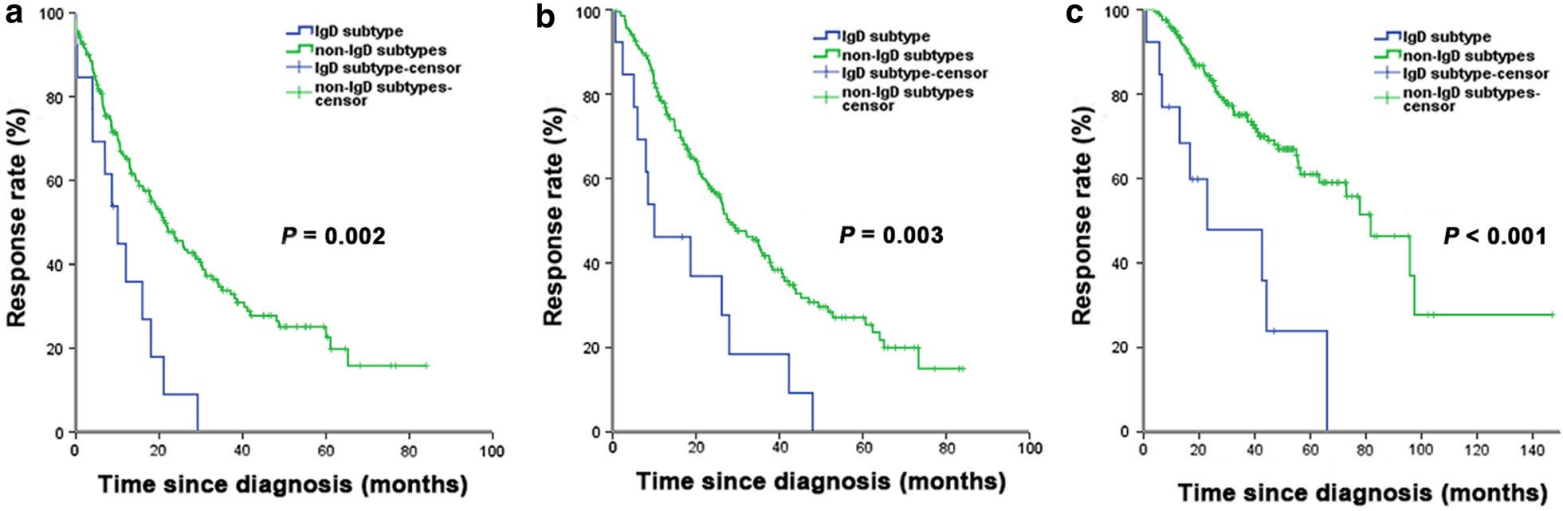
**a** Duration of response in patients with IgD subtype or non-IgD subtypes of multiple myeloma, **b** Kaplan–Meier progression-free survival curves of patients with IgD subtype or non-IgD subtypes of multiple myeloma, **c** Kaplan–Meier overall survival curves of patients with IgD subtype or non-IgD subtypes of multiple myeloma

Lastly, we analyzed the other risk factors that might have affected the prognoses of the investigated MM patients (Additional file 1: Table S1). Univariate analyses showed that, besides the IgD subtype, patients with high β_2_M level, elevated LDH level, having extramedullary infiltration, ISS stage III, 13q deletion, 1q21 amplification, IGH rearrangement, and abnormal sFLC ratio had shorter OS compared with their counterparts (Additional file 2: Figure S1). Multivariate analyses showed that IgD subtype was an independent adverse factor for both PFS (*P *= 0.009) and OS (*P *= 0.001) (Table 5).

**Table 5:** Multivariate analysis for PFS and OS of 216 patients with multiple myeloma

Variable	PFS			OS		
	HR	95% CI	*P* value	HR	95% CI	*P* value
LDH > 250 U/L	1.494	1.020–2.189	0.039	2.791	1.692–4.604	< 0.001
ISS stage III	1.298	1.032–1.633	0.026	1.533	1.096–2.145	0.013
IgD subtype	2.221	1.221–4.040	0.009	3.506	1.687–7.285	0.001
1q21 amplification	–	–	–	1.949	1.179–3.224	0.009
IGH rearrangement	1.615	1.260–2.071	< 0.001	1.485	1.165–1.895	0.001
Extramedullary infiltration	2.084	1.421–3.055	< 0.001	3.692	2.199–6.200	< 0.001

*PFS* progression-free survival, *OS* overall survival, *HR* hazard ratio, *CI* confidence interval, *LDH* lactate dehydrogenase, *ISS* international staging system, *IgD* immunoglobulin D, *IGH* immunoglobulin heavy chain gene

The synthesis rate of IgD is very low in patients bearing the IgD subtype, which often leads to missed diagnosis [2]. Among the 216 MM patients, 13 had IgD subtype (6%). This proportion was similar to another report in China (5.4%) [8] but was slightly higher than that observed in western countries (1%–2%) [2]. Nevertheless, the proportion of IgD subtype may still be underestimated, therefore, identifying and understanding this disease is extremely essential.

It has been reported that the IgD subtype of MM occurred more often in young patients, with a median age of 52 to 60 years. Moreover, it was found to be associated with higher β_2_M, extramedullary involvement, secondary systemic amyloidosis, a λ light chain bias (IgD myeloma is characterized by the presence of a predominance of λ over K light chain type), renal failure, and short survival [3]. The clinical characteristics of our patients were similar to the results of the above-mentioned studies.

In addition, we interestingly found that patients with the IgD subtype demonstrated significant sFLC ratio abnormalities at baseline and during disease relapse, especially in the 46.2% of patients with disease relapse. This finding may be conducive to assess the disease progression and to identify early relapse for timely intervention. Moreover, as compared to the IgD subtype patients with abnormal sFLC ratio, those with normal sFLC ratio had a numerically superior OS (42.56 months vs. 5.7 months, *P *= 0.057, Additional file 3: Figure S2). There was noted solely as a tendency, which may have been most likely due to the small cohort size.

High-risk cytogenetic abnormalities in MM patients with IgD subtype have been reported to range from 30 to 50%. In our study, 1q21 amplification was observed in 75% of patients with IgD subtype. It was reported that the overexpression of genes mapping to 1q21 could regulate the growth and resistance of MM to drugs, and result in increasing risk of early death of the patients [10]. Recent research has also found that the adverse effects due to 1q21 amplification on prognosis persisted even after removal of other identified high-risk cytogenetic changes, including p53 deletion, t(4;14), t(14;16), and t(14;20) [11]. Therefore, the association of IgD MM with a high rate of 1q21 amplification might contribute to poor outcomes. Perhaps it could also explain why patients with IgD subtype and non-IgD subtypes had similar response rates, but different duration of response.

Researchers have tried different ways to improve the prognosis of patients with IgD subtype all along. In 2005, Wechalekar et al. [5] suggested that the mean OS of IgD subtype patients could be prolonged after ASCT when compared with chemotherapy (5.1 years vs. 2 years, respectively, *P* = 0.090). In 2014, Zagouri et al. [12] reported a median OS of 51.5 months in 31 IgD subtype patients in Greece, which was the longest survival treated with chemotherapy reported until now. However, in Asia, the data seemed less consistent. In 2008 and 2010, two studies from Korea reported poor outcomes for the IgD subtype patients following ASCT or conventional chemotherapy, with the median OS of 12 and 18.5 months [4, 7]. In 2015, a report from China showed that the median OS and PFS of IgD subtype were 24 and 15.5 months, respectively and no difference in OS was found among the bortezomib-only group, the non-bortezomib group, and the bortezomib + ASCT group [8]. Here, our patients with IgD subtype showed similar median OS (22.9 months) and PFS (10 months) as to these studies from Asia. Though patients with non-IgD subtypes had a favourable median OS of 81.7 months in our study, in the era of novel agents, the survival of patients with IgD subtype still cannot be improved from the new drugs, unlike the non-IgD subtypes. Recently, a case report on a patient with IgD subtype who was refractory to at least 5 different chemotherapy regimens had shown very good partial response to daratumumab (anti-CD38 monoclonal antibody) [13]. We expect that additional agents with novel mechanism including histone deacetylation, target of surface receptors, and chimeric antigen receptor T Cell immunotherapy would improve the survival of IgD subtype patients.

In summary, the IgD subtype was found to be an independent adverse risk factor for prognosis. MM patients with IgD subtype presented with a more aggressive disease course and had shorter survival with chemotherapy as compared to the non-IgD subtypes, even in this era of novel agents. Considering the rarity of this subtype, international collaborative studies are suggested to confirm our findings and further elucidate the underlying mechanisms for developing potent therapeutic approaches.

## Supplementary information

**Additional file 1: Table S1.** Univariate analysis for PFS and OS of 216 patients with multiple myeloma.

**Additional file 2: Figure S1.** Kaplan–Meier overall survival curves of patients with multiple myeloma. a: Survival curves of patients stratified according to β_2_M, ≤ 5.5 mg/L vs. > 5.5 mg/L (*P *= 0.002); b: Survival of patients according to LDH, ≤ 250U/L vs. > 250U/L (*P *< 0.001); c: Survival of patients with and without extramedullary infiltration (*P* < 0.000); d: Survival of patients with and without ISS stage III (*P *= 0.028); e: Survival of patients with and without 13q deletion (*P *= 0.003); f: Survival of patients with and without 1q21 amplification (*P *< 0.000); g: Survival of patients with and without different IGH rearrangement [non-IGH rearrangement vs. t(4;14) vs. t(11;14) vs. t(14;16), *P *< 0.000]; h: Survival of patients with and without abnormal sFLC ratios (*P *= 0.035).

**Additional file 3: Figure S2.** Overall survival in IgD myeloma patients with and without abnormal sFLC ratio.

## Data Availability

The dataset used or analyzed during the current study are available from the corresponding author on reasonable request.

## Abbreviations

ASCT: autologous stem cell transplantation
AL amyloidosis: amyloid light-chain amyloidosis
β_2_M: high β_2_ microglobulin
CR: complete response
CI: confidence interval
HR: hazard ratio
IgD MM: immunoglobulin D multiple myeloma
IGH: immunoglobulin heavy chain gene
IMiD: immunomodulatory drug
ISS: international staging system
LDH: lactate dehydrogenase
OS: overall survival
PD: progressive disease
PFS: progression-free survival
PR: partial response
sFLC: serum-free light chain
sCR: strict complete response
SD: stable disease
VGPR: very good partial response
