# Supplementary material for: Poor outcomes of immunoglobulin D multiple myeloma patients in the era of novel agents: a single-center experience
Source: Cancer Commun (Lond). 2019 Sep 27;39:51. doi: 10.1186/s40880-019-0395-3 (PMC6764140; doi:10.1186/s40880-019-0395-3)
Supplement: Supplementary file 1 — Additional file 1: Table S1. Univariate analysis for PFS and OS of 216 patients with multiple myeloma. [file 40880_2019_395_MOESM1_ESM.docx]

Additional file 1: Table S1. Univariate analysis for PFS and OS of 216 patients with multiple myeloma

| Variable | PFS | | | OS | | |
| --- | --- | --- | --- | --- | --- | --- |
|  | HR | 95% CI | *P* value | HR | 95% CI | *P* value |
| β_2_Μ > 5.5 mg/L | - | - | 0.172 | 2.346 | 1.331-4.135 | 0.003 |
| LDH > 250 U/L | 1.510 | 1.037-2.199 | 0.031 | 2.918 | 1.810-4.705 | < 0.001 |
| ISS stage III | - | - | 0.058 | 1.448 | 1.068-1.963 | 0.028 |
| IgD subtype | 2.441 | 1.346-4.428 | 0.003 | 3.819 | 1.885-7.737 | < 0.001 |
| 13q deletion | - | - | 0.815 | 2.059 | 1.256-3.376 | 0.004 |
| 1q21 amplification | - | - | 0.054 | 2.557 | 1.570-4.163 | < 0.001 |
| IGH rearrangement | 1.312 | 1.079-1.596 | 0.006 | 1.615 | 1.260-2.071 | < 0.001 |
| Abnormal sFLC ratio | - | - | 0.095 | 2.734 | 11.220-6.127 | 0.035 |
| Extramedullary infiltration | 1.765 | 1.230-2.532 | 0.002 | 2.469 | 1.535-3.969 | 0.001 |

Abbreviations: PFS = progression-free survival; OS = overall survival; HR = hazard ratio; β_2_M = β_2_ microglobulin; LDH = lactate dehydrogenase; ISS = international staging system; IgD = immunoglobulin D; IGH = immunoglobulin heavy chain gene; sFLC = serum free light chain.
