# Supplementary figures and images for: Poor outcomes of immunoglobulin D multiple myeloma patients in the era of novel agents: a single-center experience
Source: Cancer Commun (Lond). 2019 Sep 27;39:51. doi: 10.1186/s40880-019-0395-3 (PMC6764140; doi:10.1186/s40880-019-0395-3)

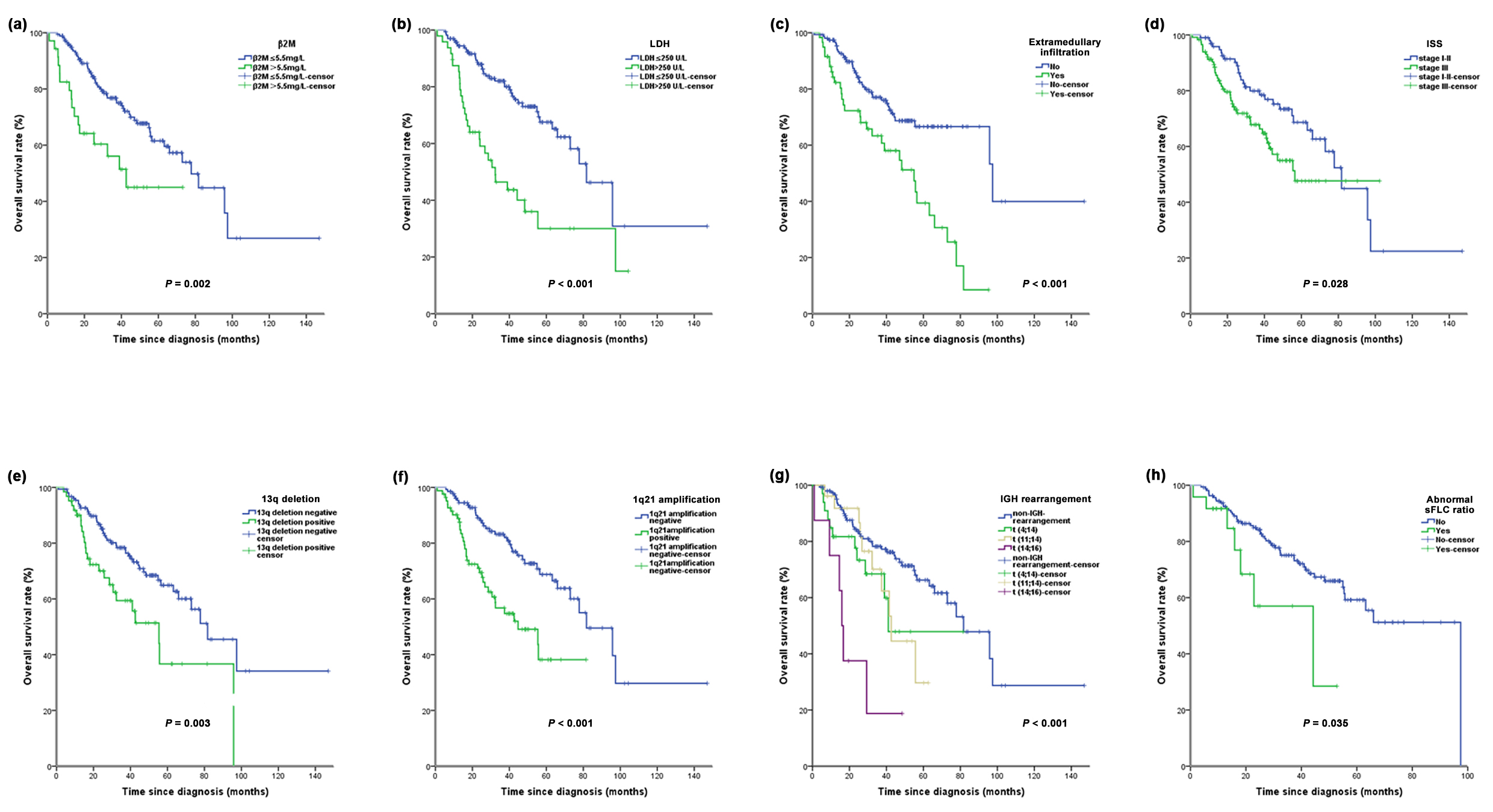

Supplement: Supplementary file 2 — Additional file 2: Figure S1. Kaplan–Meier overall survival curves of patients with multiple myeloma. a: Survival curves of patients stratified according to β2M, ≤ 5.5 mg/L vs. > 5.5 mg/L (P = 0.002); b: Survival of patients according to LDH, ≤ 250U/L vs. > 250U/L (P < 0.001); c: Survival of patients with and without extramedullary infiltration (P < 0.000); d: Survival of patients with and without ISS stage III (P = 0.028); e: Survival of patients with and without 13q deletion (P = 0.003); f: Survival of patients with and without 1q21 amplification (P < 0.000); g: Survival of patients with and without different IGH rearrangement [non-IGH rearrangement vs. t(4;14) vs. t(11;14) vs. t(14;16), P < 0.000]; h: Survival of patients with and without abnormal sFLC ratios (P = 0.035). [file 40880_2019_395_MOESM2_ESM.jpg]

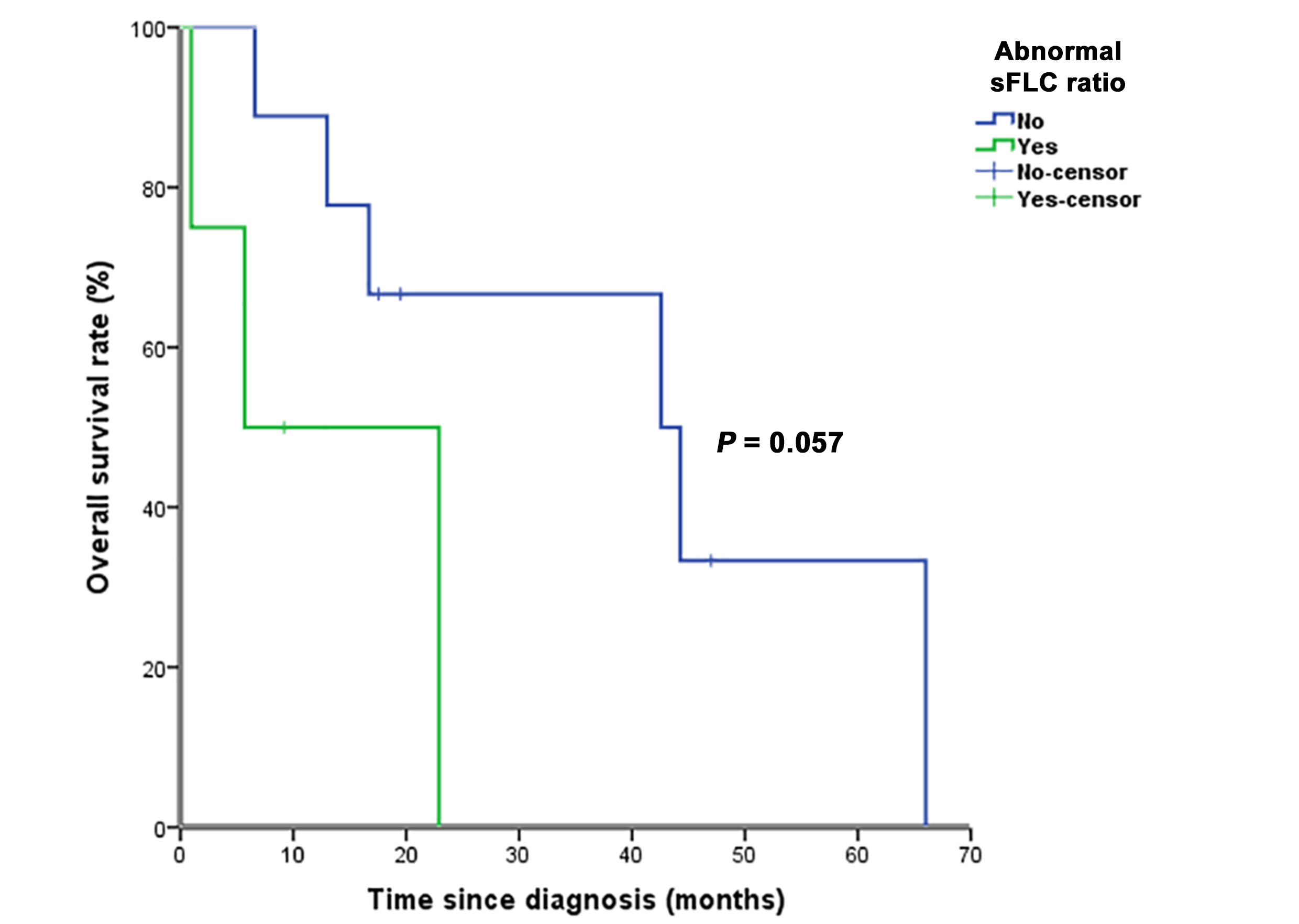

Supplement: Supplementary file 3 — Additional file 3: Figure S2. Overall survival in IgD myeloma patients with and without abnormal sFLC ratio. [file 40880_2019_395_MOESM3_ESM.jpg]
